# Rectal Lymphogranuloma Venereum, Buenos Aires, Argentina

**DOI:** 10.3201/eid2503.180600

**Published:** 2019-03

**Authors:** Laura Svidler López, Luciana La Rosa, Andrea Carolina Entrocassi, Dolores Caffarena, Brian Santos, Marcelo Rodríguez Fermepin

**Affiliations:** Hospital Fernández,. Buenos Aires, Argentina (L. Svidler López, B. Santos);; Centro Privado de Cirugía y Coloproctología, Buenos Aires (L. La Rosa, D. Caffarena);; Universidad de Buenos Aires, Buenos Aires (A.C. Entrocassi, M. Rodriquez Fermepin)

**Keywords:** Lymphogranuloma venereum, proctitis, HIV, sexually transmitted infections, men who have sex with men, Chlamydia trachomatis, bacteria, Buenos Aires, Argentina

## Abstract

Among 34 men with proctitis in Buenos Aires, Argentina, 16 (47%) had *Chlamydia trachomatis* infection, 11 (68.8%) of which were biovar lymphogranuloma venereum. The outbreak was probably local, as in Europe. In Argentina, lymphogranuloma venereum should be a suspected cause of proctitis in HIV-infected men who have had unprotected anal sex with men.

Lymphogranuloma venereum (LGV) is a sexually transmitted infection caused by *Chlamydia trachomatis* serovars L1, L2, or L3 and their variants. LGV has been considered endemic to Asia, Africa, and the tropical region of South America. Over the past 2 decades, case reports of LGV in Argentina have been sporadic and regarding only patients who acquired the infection abroad.

In the Netherlands in 2003, an outbreak of rectal LGV among men who have sex with men (MSM), mainly HIV infected, was reported (*1*). This report was followed by many other reports from other developed countries (*2*,*3*).

LGV has been traditionally described as causing inflammation and swelling of the inguinal lymph nodes and also involving the rectum, causing acute proctitis, particularly among HIV-infected MSM (*4*). Since 2015, some clinicians in Argentina have suspected LGV in certain patients with proctitis (regardless of association with inflammatory tumors) in which *C. trachomatis* has been detected but not genotyped. Thus, we conducted a prospective study to assess the *C. trachomatis* genotypes as the causative agent of infectious proctitis in Buenos Aires, Argentina. Our study was conducted in a private practice and a public hospital, under a protocol previously approved by the hospital’s ethics committee (no. 201723).

From September 1, 2017, through February 1, 2018, we included in our study every man who visited either the private or public study site and who had rectal signs or symptoms of proctitis and had not taken antimicrobial drugs in the previous month. None of the included patients was referred by a previously included patient. Each participant signed an informed consent form. 

Over the first 5 months, we obtained a rectal swab sample from 34 men on their first visit. To detect *C. trachomatis*, we extracted DNA from the samples by using real-time PCR targeting a cryptic plasmid fragment (Alert PCR; ELITech Molecular Diagnostics, https://www.elitechgroup.com). Positive samples were genotyped by *ompA*-based PCR restricted fragment length polymorphism (*5*).

Of the 34 samples analyzed, 16 were positive for *C. trachomatis*; 11 were identified as genotype L2 and 5 as genotypes D, F, or J. All participants reported having engaged in unprotected receptive anal sex in Argentina, except for 1 who had had receptive anal sex while in Mexico. None declared having traveled to an LGV-endemic area. Mean age was 31.63 years (range 22–43 years). All but 1 were HIV positive, and 5 had another sexually transmitted infection (1 had gonorrhea, 1 had syphilis and gonorrhea, 1 had syphilis only, and 2 had viral condylomas). The signs and symptoms of proctitis included rectal pain; tenesmus; and a discharge that was mucous, crystalline, hematic, or purulent. Endoscopic appearances of proctitis were variable, from mild to severe. Three patients had perianal lesions, 1 had a fistula, and 2 had ulcers. One patient had severe proctitis with rectal stenosis and was suspected of having had inflammatory bowel disease (IBD) for 1 year at the time of the first interview.

All patients were prescribed doxycycline for 3 weeks as recommended (*4*), resulting in complete resolution of proctitis and perianal ulcers. Two patients with chronic complications required additional treatments (surgical resolution of fistula and endoscopic dilatation of stenosis), resulting in complete resolution.

We observed a high prevalence (47%) of chlamydial infection within the studied group (16/34); the most frequent biovar was LGV (68.8% of the *Chlamydia*-infected patients). The cases included in this series were detected within a short period, which suggests a local outbreak. As in the series in Europe, the patients in Argentina were MSM, almost all HIV infected, who reported having had unprotected anal sex (*6*,*7*).

Signs and symptoms from most patients did not suggest LGV: 7 of the 11 with genotype L2 had mild or moderate proctitis similar to non-LGV infection. In 1 patient, severe proctitis mimicked an inflammatory tumor; in another, IBD. Proctitis is a nonspecific manifestation with diverse origins, for which clinical or endoscopic findings are insufficient to determine etiology, as observed in this case series. Also, because in about one quarter of patients with LGV infections the symptoms are mild or absent, diagnosis can be missed or late (*8*,*9*). Thus, given this evidence and the similarities between LGV and IBD, gastroenterologists should consider LGV as a differential diagnosis for patients with proctitis, especially HIV-infected MSM. Because the clinical assessment is not specific and quite inconclusive on which to base a diagnosis with certainty, genotyping is needed to indicate the proper patient care, management, and treatment. Including *C. trachomatis* typing as a routine diagnostic step also helps avoid chronic complications, stop the transmission chain (*3*,*4*,*10*), and provide useful information for epidemiologic surveillance. We conclude that in Buenos Aires, Argentina, as well as in countries in Europe, rectal LGV should be suspected as a cause of proctitis in HIV-infected MSM with a history of unprotected anal sex.

## References

[1] Nieuwenhuis RF, Ossewaarde JM, Götz HM, Dees J, Thio HB, Thomeer MG, et al. Resurgence of lymphogranuloma venereum in Western Europe: an outbreak of *Chlamydia trachomatis* serovar l2 proctitis in The Netherlands among men who have sex with men. Clin Infect Dis. 2004;39:996–1003. 10.1086/423966 15472852

[2] Rodríguez-Domínguez M, Puerta T, Menéndez B, González-Alba JM, Rodríguez C, Hellín T, et al. Clinical and epidemiological characterization of a lymphogranuloma venereum outbreak in Madrid, Spain: co-circulation of two variants. Clin Microbiol Infect. 2014;20:219–25. 10.1111/1469-0691.12256 23730727

[3] Pallawela SN, Sullivan AK, Macdonald N, French P, White J, Dean G, et al. Clinical predictors of rectal lymphogranuloma venereum infection: results from a multicentre case-control study in the U.K. Sex Transm Infect. 2014;90:269–74. 10.1136/sextrans-2013-051401 24687130 PMC4033117

[4] de Vries HJ, Zingoni A, Kreuter A, Moi H, White JA; European Branch of the International Union against Sexually Transmitted Infections; European Academy of Dermatology and Venereology; European Dermatology Forum; European Society of Clinical Microbiology and Infectious Diseases; Union of European Medical Specialists; European Centre for Disease Prevention and Control; European Office of the World Health Organisation. 2013 European guideline on the management of lymphogranuloma venereum. J Eur Acad Dermatol Venereol. 2015;29:1–6. 10.1111/jdv.12461 24661352

[5] Lan J, Walboomers JM, Roosendaal R, van Doornum GJ, MacLaren DM, Meijer CJ, et al. Direct detection and genotyping of *Chlamydia trachomatis* in cervical scrapes by using polymerase chain reaction and restriction fragment length polymorphism analysis. J Clin Microbiol. 1993;31:1060–5. 8099080 10.1128/jcm.31.5.1060-1065.1993 PMC262880

[6] Foschi C, Marangoni A, D’Antuono A, Nardini P, Compri M, Bellavista S, et al. Prevalence and predictors of Lymphogranuloma venereum in a high risk population attending a STD outpatients clinic in Italy. BMC Res Notes. 2014;7:225. 10.1186/1756-0500-7-225 24716676 PMC3984434

[7] Latini A, Zaccarelli M, Paglia MG, Donà MG, Giglio A, Moretto D, et al. Inguinal and anorectal Lymphogranuloma Venereum: a case series from a sexually transmitted disease center in Rome, Italy. BMC Infect Dis. 2017;17:386. 10.1186/s12879-017-2484-8 28577539 PMC5455085

[8] Saxon C, Hughes G, Ison C; UK LGV Case-Finding Group. Asymptomatic lymphogranuloma venereum in men who have sex with men, United Kingdom. Emerg Infect Dis. 2016;22:112–6. 10.3201/EID2201.141867 26691688 PMC4696683

[9] Van der Bij AK, Spaargaren J, Morré SA, Fennema HS, Mindel A, Coutinho RA, et al. Diagnostic and clinical implications of anorectal lymphogranuloma venereum in men who have sex with men: a retrospective case-control study. Clin Infect Dis. 2006;42:186–94. 10.1086/498904 16355328

[10] Quint KD, Bom RJ, Quint WG, Bruisten SM, van der Loeff MF, Morré SA, et al. Anal infections with concomitant *Chlamydia trachomatis* genotypes among men who have sex with men in Amsterdam, the Netherlands. BMC Infect Dis. 2011;11:63. 10.1186/1471-2334-11-63 21401939 PMC3068958

